# Rapidly progressive mitral valve disease from non-bacterial thrombotic endocarditis to mitral stenosis in systemic lupus erythematosus: a case report

**DOI:** 10.1093/ehjcr/ytaf098

**Published:** 2025-02-25

**Authors:** Saaya Ichikawa-Ogura, Yasuhide Mochizuki, Eiji Toyosaki, Hiroto Fukuoka, Toshiro Shinke

**Affiliations:** Division of Cardiology, Showa University School of Medicine, 1-5-8 Hatanodai, Shinagawa-ku, Tokyo 142-8555, Japan; Division of Cardiology, Showa University School of Medicine, 1-5-8 Hatanodai, Shinagawa-ku, Tokyo 142-8555, Japan; Division of Cardiology, Showa University School of Medicine, 1-5-8 Hatanodai, Shinagawa-ku, Tokyo 142-8555, Japan; Division of Cardiology, Showa University School of Medicine, 1-5-8 Hatanodai, Shinagawa-ku, Tokyo 142-8555, Japan; Division of Cardiology, Showa University School of Medicine, 1-5-8 Hatanodai, Shinagawa-ku, Tokyo 142-8555, Japan

**Keywords:** Libman–Sacks endocarditis, Non-bacterial thrombotic endocarditis, Systemic lupus erythematosus, Antiphospholipid syndrome, Mitral stenosis, Case report

## Abstract

**Background:**

Libman–Sacks endocarditis), a non-bacterial thrombotic endocarditis (NBTE) linked to systemic lupus erythematosus (SLE) and antiphospholipid syndrome (APS), typically causes valve regurgitation and embolism but can rarely mimic rheumatic mitral stenosis (MS).

**Case summary:**

This case involves a 59-year-old woman with a history of APS and SLE who presented with worsening dyspnoea and congestive heart failure. Initially, severe mitral regurgitation (MR) due to NBTE resolved with vitamin K antagonist therapy, yet she subsequently developed significant MS with commissural fusion, a rheumatic-like feature. Despite stable SLE activity, echocardiography revealed severe MS with high pulmonary pressures, warranting surgical valve replacement. Intraoperative findings confirmed rheumatic-like degeneration, but the patient experienced a fatal cerebral infarction post-surgery, likely due to APS.

**Discussion:**

This case highlights the progression of NBTE-related MR to rheumatic-like MS in an SLE patient with APS, an unusual clinical course. It underscores the importance of echocardiographic monitoring in similar cases, as chronic inflammatory changes in APS might mimic rheumatic pathology, necessitating vigilant management and timely intervention.

**Learning points**

- Mitral valve involvement in systemic lupus erythematosus and antiphospholipid syndrome (APS) can lead to rare valvular manifestations such as non-bacterial thrombotic endocarditis (NBTE) and rheumatic-like mitral stenosis (MS), which may progress despite controlled disease activity.
- Anticoagulation therapy with vitamin K antagonists can be effective in managing NBTE, improving mitral regurgitation, but may not prevent the development of other valvular complications such as rheumatic-like MS in autoimmune diseases.
- There is no clear evidence to determine whether transcatheter commissurotomy or surgical valve replacement is the better option for MS caused by NBTE associated with APS.

## Introduction

Libman–Sacks endocarditis (LSE), described in 1924 by Emanuel Libman and Benjamin Sacks, is a cardiac manifestation of systemic lupus erythematosus (SLE). It presents as sterile vegetation on mitral and aortic valves and is a type of non-bacterial thrombotic endocarditis (NBTE) linked to SLE and antiphospholipid syndrome (APS). Key complications include systemic embolism and regurgitation from incomplete valvular coaptation. Rarely, LSE mimics rheumatic valvular disease, causing stenosis through inflammatory commissural fusion.^1–4^ We present a case where anticoagulation effectively treated NBTE-associated mitral regurgitation (MR), but rheumatic-like mitral stenosis (MS) developed over four years, necessitating surgical valve replacement.

## Summary figure

**Table:** 

Date	Event	Figure
15 years old	Diagnosis of antiphospholipid syndrome	
18 years old	Diagnosis of systemic lupus erythematosus	
	Steroid therapy initiated	
55 years old (2019/2)	Dyspnoea on exertion appeared	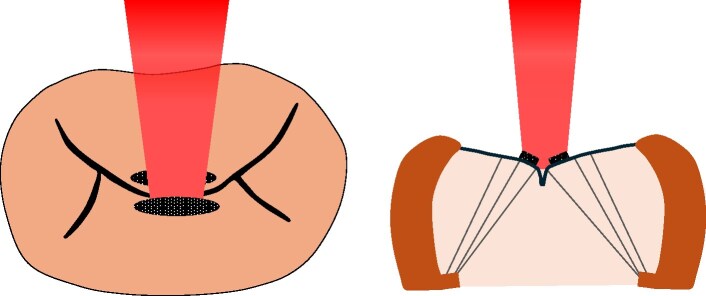
	Echocardiography demonstrated severe mitral regurgitation (MR) with non-bacterial thromboendocarditis (NBTE)	
	Anticoagulation therapy (warfarin potassium) started	
55 years old (2019/10)	MR improved to mild grade	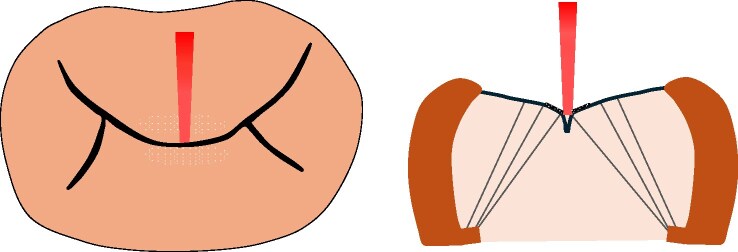
	NBTE had almost disappeared	
58 years old (2022/11)	Echocardiography revealed mild MR and newonset mitral stenosis (MS)	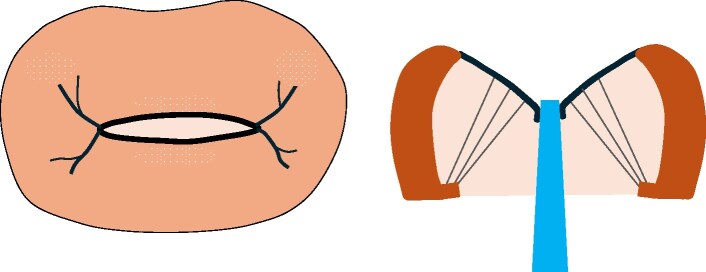
59 years old (2023/11)	Hospitalization due to congestive heart failure	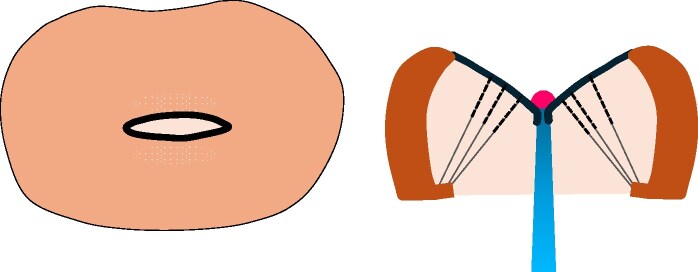
	Elevated brain natriuretic peptide level of 895 pg/mL	
	Echocardiography showed severe MS and pulmonary hypertension	
59 years old (2023/11)	Surgical mitral valve replacement (27-mm MITRIS RESILIA)	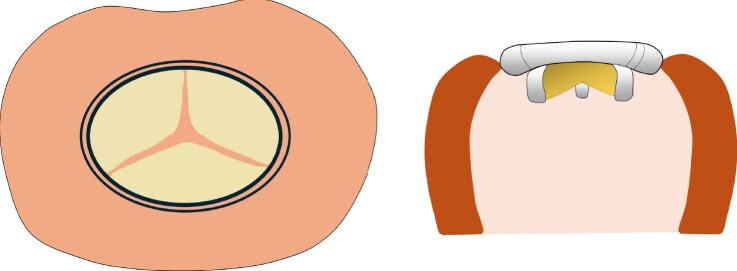

## Case presentation

**Figure 1:**
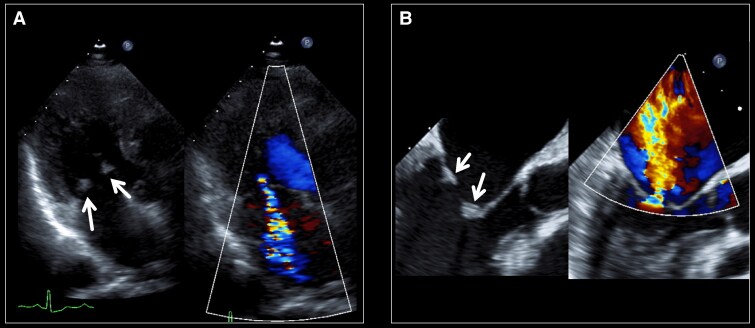
Transthoracic (*A*) and transoesophageal (*B*) echocardiography in February 2019 showing non-bacterial thrombotic endocarditis (white arrow) and severe mitral regurgitation.

**Figure 2:**
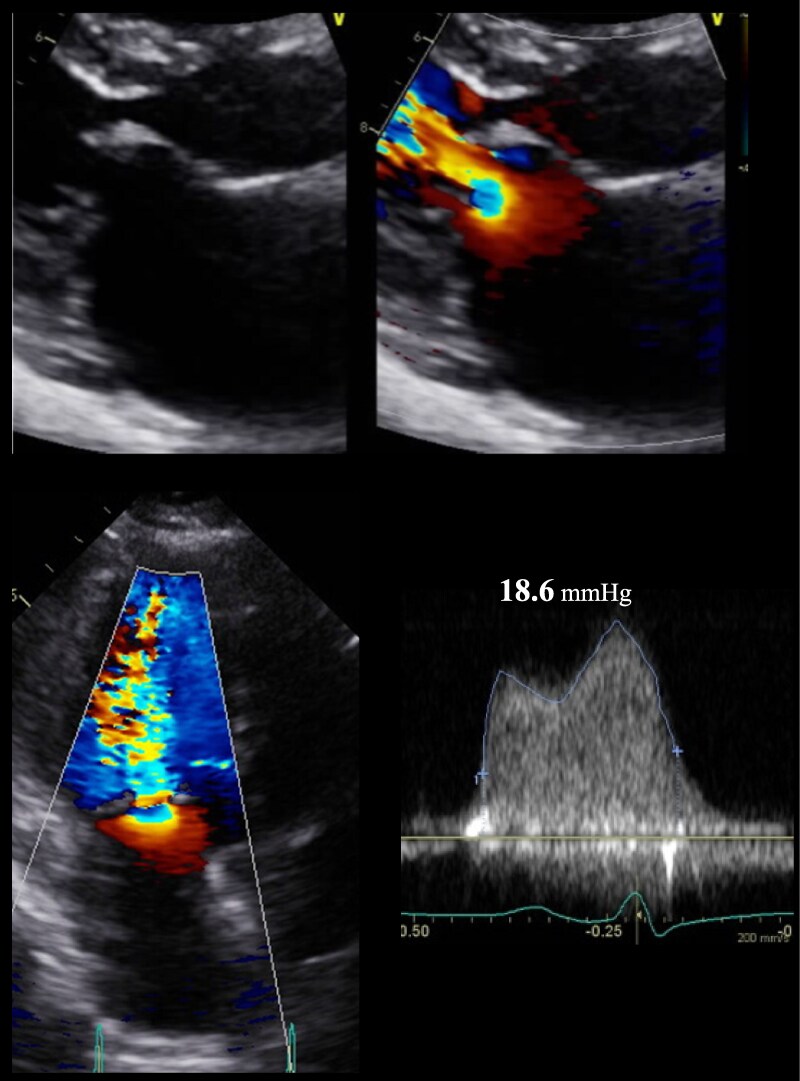
Transthoracic echocardiography at admission for congestive heart failure revealed severe mitral valve stenosis, with a mean transvalvular pressure gradient of 18.6 mmHg across the mitral valve.

**Figure 3:**
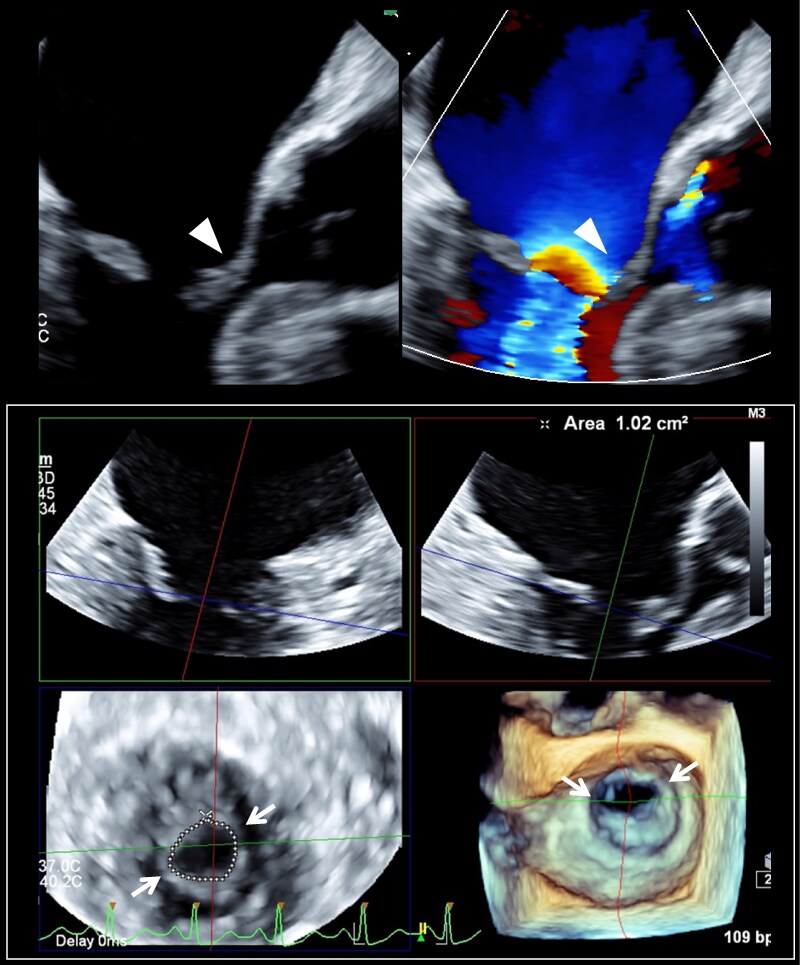
Transoesophageal echocardiography during hospitalization for congestive heart failure in November 2023 demonstrated severe mitral valve stenosis. The mitral valve leaflets showed marked thickening with doming of the anterior leaflet (arrowhead), and commissural fusion (white arrow) with severe restriction of valve opening was evident. The mitral valve area was calculated as 1.02 cm² using three-dimensional multiplanar reconstruction analysis.

**Figure 4:**
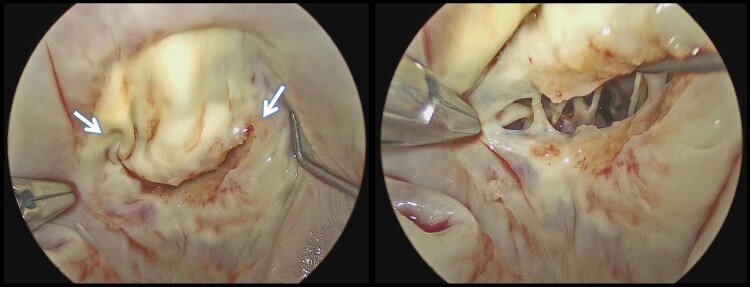
Intraoperative view showing bilateral commissural fusion (arrow), with marked thickening of both mitral valve leaflets and chordae tendineae.

A 59-year-old woman was diagnosed with APS at age 15 and SLE at age 18. She later developed central nervous system lupus, recurrent miscarriage, thrombocytopaenia-related irregular vaginal bleeding, and lupus nephritis. Her history included steroid-induced diabetes, bronchial asthma, and avascular necrosis of the femoral head. At age 55, she presented with exertional dyspnoea. Transthoracic echocardiography (TTE) and transoesophageal echocardiography (TOE) revealed severe MR and a 5 mm mass on the left atrial side of both mitral valve leaflets (*Figure 1*). Transthoracic echocardiography revealed left ventricular ejection fraction (LVEF) of 62%, left atrial volume of 40 mL/m^2^. The mean pressure gradient (MPG) through the mitral valve was 5.3 mmHg, and the mitral valve area (MVA) calculated using the pressure half-time (PHT) method was 2.3 cm², suggesting mild MS. Two sets of blood cultures were negative, and she was diagnosed with NBTE associated with LSE. Oral anticoagulation therapy with vitamin K antagonist (VKA) was initiated via heparin bridging therapy, and prothrombin time and international normalized ratio was stably maintained at around 2.0 with 2 mg. After 8 months of sustained therapy, the NBTE resolved completely, and MR improved to a mild grade (see Supplementary material online, *Movie S1*). At age 58, TTE revealed progression to moderate MS with MPG of 7.5 mmHg, MVA of 1.1 cm² by PHT, and 1.4 cm² by planimetry, along with bilateral commissural fusion, while MR remained mild. Brain natriuretic peptide (BNP) levels remained relatively low at ∼30 pg/mL, and SLE disease activity was well-controlled. At age 59, she presented with a 1-month history of persistent dyspnoea and was admitted for congestive heart failure (HF). Physical examination revealed bilateral lower limb oedema, and auscultation detected wheezing. Electrocardiography revealed sinus rhythm with a heart rate of 105 beats/min. Chest radiography demonstrated pulmonary congestion and bilateral pleural effusions. As shown in *Table 1*, the laboratory data revealed elevated inflammatory markers and a marked increase in BNP levels. The patient had been prescribed daily oral medications in the outpatient setting, including 2 mg warfarin potassium, low-dose aspirin, 16 mg prednisolone, and 1250 mg mycophenolate mofetil daily, which were continued after admission. After approximately one week of treatment for congestive HF with intravenous furosemide 20 mg/day and oral tolvaptan 3.75 mg, the congestion improved. On admission, TTE showed a preserved LVEF of 60% and a left atrial volume index of 42 mL/m². The MVA with bilateral commissural fusion was 0.86 cm² (calculated by continuity equation), and the MPG was markedly elevated at 18.6 mmHg, confirming severe MS (*Figure 2*, Supplementary material online, *Movie S2*). Transoesophageal echocardiography performed after HF treatment confirmed thickened mitral leaflets, complete resolution of the NBTE, bilateral commissural fusion, and diastolic doming (*Figure 3* and Supplementary material online, *Movie S3*). The mitral valve area was calculated as 1.02 cm² using three-dimensional multiplanar reconstruction analysis. No significant valvular disease other than MS, thrombi, or vegetation was detected. Cardiac catheterization revealed an elevated pressure gradient of 25 mmHg between the pulmonary artery wedge pressure and the left ventricle, suggesting increased left atrial pressure. The mean pulmonary arterial pressure was 30 mmHg, indicating post-capillary pulmonary hypertension. These findings confirmed that MS was clinically significant. Given the high surgical risk with an STS score of 27.5 for mitral valve replacement, percutaneous transvenous mitral commissurotomy was considered a less invasive alternative. However, the severe commissural fusion and the history of NBTE raised concerns about procedural embolism. After a heart team conference, surgical valve replacement was chosen. The operation was performed two months after the HF hospitalization. Vitamin K antagonist was discontinued three days before surgery, and strict anticoagulation management was achieved during the perioperative period using intravenous heparin, monitored with the HMS PLUS heparin concentration measurement system (Medtronic). A 27-mm MITRIS RESILIA bioprosthetic valve was implanted. Intraoperative findings were consistent with TOE results, revealing rheumatic degeneration characterized by bilateral commissural fusion and leaflet thickening (*Figure 4*). Despite rigorous perioperative management, including intravenous heparin and HF control, the patient developed a left middle cerebral artery infarction, presumably due to APS, and died on postoperative Day 180.

**Table 1:** Laboratory data on admission for heart failure

Parameter	Result	Normal range (for an adult woman)
White blood cell count, /μL	12 700	4000–11 000
Haemoglobin, g/dL	7.5	12.1–15.1
C-reactive protein, mg/dL	3.1	<0.1
BNP, pg/mL	895	≤18.4
Serum creatinine, mg/dL	1.74	0.5–0.9
Creatinine clearance, mL/min	33.5	80–130
Lupus anticoagulant test	Positive	Negative
Haemolytic complement activity, U/mL	55	30–50
Anti-dsDNA antibody levels, IU/mL	0.6	<30

## Discussion

This report highlights an atypical clinical course of LSE. The patient initially presented with MR due to NBTE, which resolved completely with VKA therapy but later progressed unexpectedly to rheumatic-like MS, culminating in congestive HF (see Supplementary material online, *Movie S4*).

Non-bacterial thrombotic endocarditis presents as sterile fibrin-platelet thrombi mainly affecting mitral and aortic valves, occurring in autoimmune diseases, malignancies, and disseminated intravascular coagulation. In SLE/APS, it’s called LSE and can develop regardless of disease activity. Patients with antiphospholipid antibodies are prone to valve disease, with immune complex deposits suggesting immune-mediated inflammation.^3,5,6^ As shown by TOE in this case, NBTE features no valve destruction, with symmetrically aligned mass echoes on the mitral atrial rough zone, differing from infective endocarditis. Negative blood cultures and resolution of mass echoes with anticoagulation alone further distinguished it from infective endocarditis. Management of NBTE typically involves intravenous unfractionated or low-molecular-weight heparin to prevent embolic events. While VKA is not superior to heparin, preventing thromboembolism in APS requires high-intensity warfarin therapy.^7^ Approximately 70% of NBTE cases resolve after VKA therapy,^8^ and surgical intervention is reserved for refractory cases.^9,10^ In one reported instance, NBTE due to APS resolved, and MR severity improved following VKA therapy.^11^ In this patient, bridging intravenous heparin to VKA improved MR while mitigating procedural risk.

In SLE, MR is common, while MS is rare. Roldan *et al*.^12^ reported stenotic valvular disease in 4% of SLE patients using TOE, with no new cases or progression during follow-up exams. Similarly, Moyssakis *et al*.^4^ noted mild to moderate MS in (2.6%) of patients with SLE, with progression to severe MS during the follow-up period. A case report showed SLE/APS-associated mild MR progressing to severe MS and regurgitation after a 5-year interruption in treatment.^13^ While our case differs, showing MS progression despite optimal therapy, both cases suggest valvular disease can progress independently of SLE/APS activity and may rapidly worsen at any disease stage.

The development of rheumatic-like MS in patients with SLE remains unclear but may involve chronic inflammation leading to progressive valvular thickening, fibrosis, and scarring. Cross-reactivity in antibody specificity between rheumatic fever and APS^14^ may contribute to similar valvular degeneration, mimicking the pathology of MS.

Finally, there are no reports of successful long-term outcomes of percutaneous transvenous commissurotomy in SLE patients with APS. With risks of recurrence and perioperative thrombosis, it may be an option but is unlikely to be curative.

## Conclusion

This case presents a rare NBTE in SLE/APS, featuring MR improvement with VKA treatment, yet rapid progression to rheumatic-like MS despite controlled SLE, leading to fatal surgical intervention. This highlights the necessity of regular echocardiographic monitoring with careful attention to mitral valve morphology.

## Supplementary Material

ytaf098_Supplementary_Data

## Data Availability

The data underlying this article will be shared upon reasonable request with the corresponding authors.

## Lead author biography

Dr Saaya Ichikawa-Ogura graduated from St. Marianna University School of Medicine in Japan in 2019. Since April 2021, she has been working in the Department of Cardiology, Showa University, in Tokyo, Japan, where she conducts clinical research specializing in cardiovascular imaging of heart failure and valvular heart disease.

## Notes

**Consent**: The authors confirm that the consent for the submission and publication of this case report, including images and associated text, was obtained from the husband of the patient in line with COPE guidelines.

**Funding**: None.
